# A Statistical Approach of Background Removal and Spectrum Identification for SERS Data

**DOI:** 10.1038/s41598-020-58061-z

**Published:** 2020-01-29

**Authors:** Chuanqi Wang, Lifu Xiao, Chen Dai, Anh H. Nguyen, Laurie E. Littlepage, Zachary D. Schultz, Jun Li

**Affiliations:** 1 University of Notre Dame, Department of Applied and Computational Mathematics and Statistics, Notre Dame, IN 46556 United States; 2 The Ohio State University, Department of Chemistry and Biochemistry, Columbus, OH 43210 United States; 3 University of Notre Dame, Department of Chemistry and Biochemistry, Notre Dame, IN 46556 United States; 4 Harper Cancer Research Institute, South Bend, IN 46617 United States

**Keywords:** Biochemistry, Biological techniques, Computational biology and bioinformatics

## Abstract

SERS (surface-enhanced Raman scattering) enhances the Raman signals, but the plasmonic effects are sensitive to the chemical environment and the coupling between nanoparticles, resulting in large and variable backgrounds, which make signal matching and analyte identification highly challenging. Removing background is essential, but existing methods either cannot fit the strong fluctuation of the SERS spectrum or do not consider the spectra’s shape change across time. Here we present a new statistical approach named SABARSI that overcomes these difficulties by combining information from multiple spectra. Further, after efficiently removing the background, we have developed the first automatic method, as a part of SABARSI, for detecting signals of molecules and matching signals corresponding to identical molecules. The superior efficiency and reproducibility of SABARSI are shown on two types of experimental datasets.

## Introduction

Surface-enhanced Raman scattering (SERS) is increasingly used to identify and quantify biomolecules in complex samples^1^ because the observed Raman spectrum provides a molecular fingerprint that can be used to identify specific molecules. Advances in SERS methodology incorporating internal standards enables quantitative analysis at low concentrations. Incorporating SERS with separation methods can provide high throughput molecularly specific detection^2–6^. A significant challenge to using SERS for molecular analysis is separating the molecular signal from the large background arising from the enhancing nanostructure.

The enhanced signal originates from the interaction of analytes with the enhanced electromagnetic field from the plasmonic nanostructures^7^. These enhancements transform Raman scattering into an ultrasensitive technique that can detect single molecules^8,9^. Despite this amazing sensitivity associated with SERS, a number of challenges exist that complicate analysis and interpretation of the signals observed. First, SERS signals contain both molecular contributions and a large continuum background that is associated with the plasmonic nanostructures^10–13^. The origin of the continuum background observed in SERS spectra is not fully understood but is generally attributed to some form of plasmonic emission, which can vary with solvents, ionic strength, and changes in nanoparticle structure. At high laser intensities, molecules can photodegrade to produce broad features in the SERS spectrum, and the nanoparticles can change shape altering the emission background. Experiments that can minimize these photodegradation effects^14,15^ are important and can also promote stable backgrounds. Additionally, in solution, the molecules can diffuse away from the nanostructures and can have competitive interactions with other solution species^16^. These interactions can lead to short signal durations when the analyte can be detected^17^.

The substrate, solvent, and analytes of interest all make major contributions to a SERS spectrum^18^. Typically, the contributions to the signal from the substrate and solvent are much stronger than from the target analytes. These contributions form a strong and complicated background or baseline that must be removed so that the true signals of interest, the contributions from the analytes, can be analyzed. Background removal is a critical step for Raman data analysis^19–23^, and methods for this task are usually referred to as baseline-correction methods (BCMs).

Most BCMs process spectra one at a time by modeling and removing the background independently from each spectrum. Polynomial fitting (PF) fits the baseline by a low-order polynomial^19^ but is found to perform poorly for spectra with low signal to noise/background ratios^24,25^. Another method fits each spectrum by a smooth spline curve, but its performance can be sensitive to the choice of the positions of knots and the type of spline functions^26–28^. The wavelet transformation method^29^ transforms the spectrum into the frequency space and claims the low-frequency components to be the background. However, selecting a proper threshold between high and low frequencies can be difficult^20,24,30^. Apart from these three methods, a non-parametric method called the noise median method (NMM) was introduced for nuclear magnetic resonance data^31^, in which the baseline is first estimated by the median value in a moving window along the spectrum and then smoothed by convolving it with a Gaussian function to remove sharp discontinuities. The performance of NMM is sensitive to the choice of the size of the selected window and to the bandwidth of the Gaussian function^26^. All the above methods process spectra individually and independently, while making assumptions based on the separation between baseline and signals. Typically, these assumptions include that the baseline has low curvature and can be described by a smooth curve, while the real signals cannot be represented by a smooth shape. In fact, although the baseline mostly fluctuates less than the signals fluctuate, the baseline often still includes rapid fluctuations along the spectrum (shown in the Results section).

Some other BCMs do not include assumptions about the shape of the background at individual time points but instead assume that the shape of the background does not change over time^32,33^. They use spectra from all the time points to estimate this common shape of background. Unfortunately, this new assumption again oversimplifies the data: as we will show in the Results section, the shape of the baseline/background typically does change with time. This change is often slow but comprehensive; ignoring this change often significantly distorts the follow-up analysis, such as spectrum identification.

In this paper, we propose a new background removal method that eliminates the assumption that the baseline is unchanged over time. Instead, we allow its overall strength to change arbitrarily and its shape to change with a slow to moderate speed. We still consider multiple spectra simultaneously, so that the baseline can be of any shape at any given time point. Our method has been applied to two SERS datasets, and each time thoroughly removed the varying and complex background.

After the background from SERS data has been removed, the remaining spectra are composed of signals from different molecules, each of which comes and then goes at a certain time and also only occupies certain frequency ranges in the spectrum, as well as random, ubiquitous noises. The signals need to be picked out and then identified, either by comparing the signals to the “signature” spectrum of known molecules or by matching them across different experiments. These tasks, which are used to interpret the SERS data, have typically been done manually^5,34–36^, making them error-prone and less reproducible. In this paper, we propose the first automated and statistically rigid method for these tasks, including both the signal detection and the matching of signals across experiments. For signal detection, we created a signal filter to extract signals that are of both statistical and practical significance. For the signal matching, we propose a novel metric of similarity that takes account of the systematic differences across experiments.

**Figure 1:**
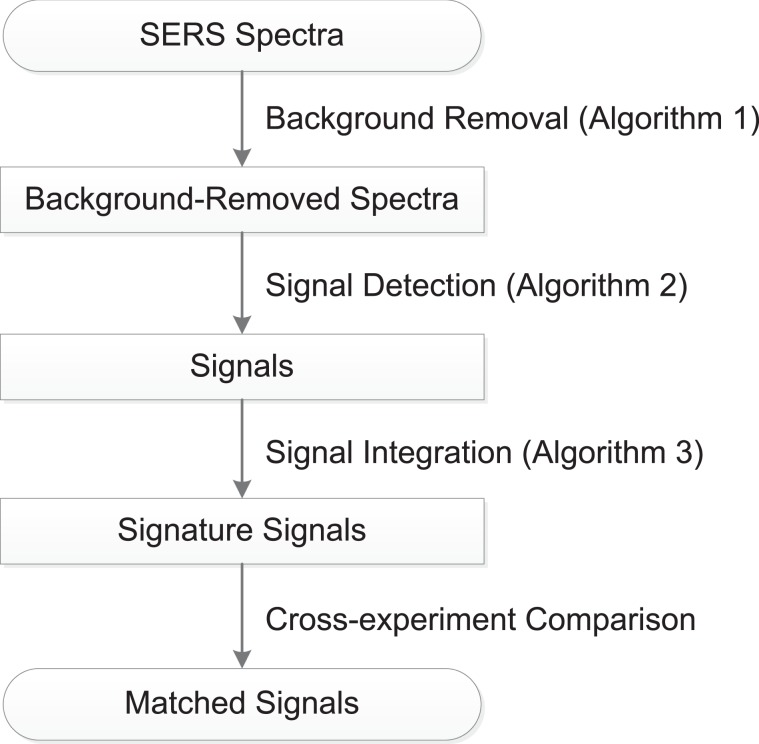
Work flow of SABARSI. The phrases in the boxes show the data used or obtained, and the phrases on the side of arrows show the operations and the algorithms used.

As Fig. 1 shows, the statistical approach we have proposed, called “SABARSI” (Statistical Approach of BAckground Removal and Spectrum Identification), forms a pipeline for SERS data analysis: background removal, signal identification, and comparison. Its performance has been evaluated here with technical replicates and across two sample types, where SABARSI not only more efficiently removed the strong and changing background, as compared to previously used BCMs, but also identified signals of interest with high reproducibility.

## Results

Two types of datasets are used to demonstrate the performance of SABARSI: a three vitamin mixture dataset and a tumor lysate dataset.

Analyzed in a previous publication^5^, the three vitamin dataset is a mixture of three B vitamins (riboflavin, thiamine, and folic acid) separated by sheath flow LC-SERS. Five technical replicates were measured and included in the dataset. In each replicate, the spectra of 1,600 frequency channels from 5,000 time points were recorded, and the time points when signals of analytes appear are summarized in Table 1.

**Table 1:** Time points of signature signals detected by SABARSI and pre-known time ranges of where signals should appear for the three vitamins in five replicates.

Replicate		1	2	3	4	5
Riboflavin	SABARSI	3570	3575	3581	3563	3541
	Pre-known	3570~3575	3573~3576	3580~3582	3563~3565	3540~3541
Thiamine	SABARSI	2793	2783	2804	2782	2761
	Pre-known	2791~2793	2780~2783	2802~2804	2779~2782	2761~2763
Folic acid	SABARSI	3641	3636	3661	3630	3643
	Pre-known	3640~3641	3634~3636	3660~3661	3629~3631	3642~3643

For the tumor lysate dataset, we generated a lysate from a mouse breast tumor and spiked the lysate with reference molecules of different concentrations. The tumor lysate dataset contains three technical replicates, where each replicate contains SERS spectra of 1,600 frequency channels collected from 6,000 time points. The experimental details are included in the Supplementary Materials.

On the three vitamin dataset that has simple, known analytes, the performance of SABARSI on background removal is compared with four existing BCMs: NMM^31^, PF^19^, iterative restricted least square (IRLS)^37^, and a constant-background correction method^4^. We also determine if SABARSI can successfully identify the pre-determined vitamins. Then, we demonstrate the performance of SABARSI on signal identification using the more complex, heterogeneous tumor dataset.

### Background removal on the three vitamin dataset: comparison with NMM, PF, and IRLS

NMM, PF, and IRLS are three BCMs that process each spectrum individually (refer to Introduction). They are publicly available in an R package called baseline^33^. Five different window sizes (10, 25, 50, 100, and 200) were used for NMM, and the best performer, window size 50, was used for comparing to the other methods. We used the default settings for PF and IRLS. For SABARSI, we set the window sizes of both time and frequency channels to be 50 to remove the background. Then we inspected the background-removed spectra of the three vitamins.

**Figure 2:**
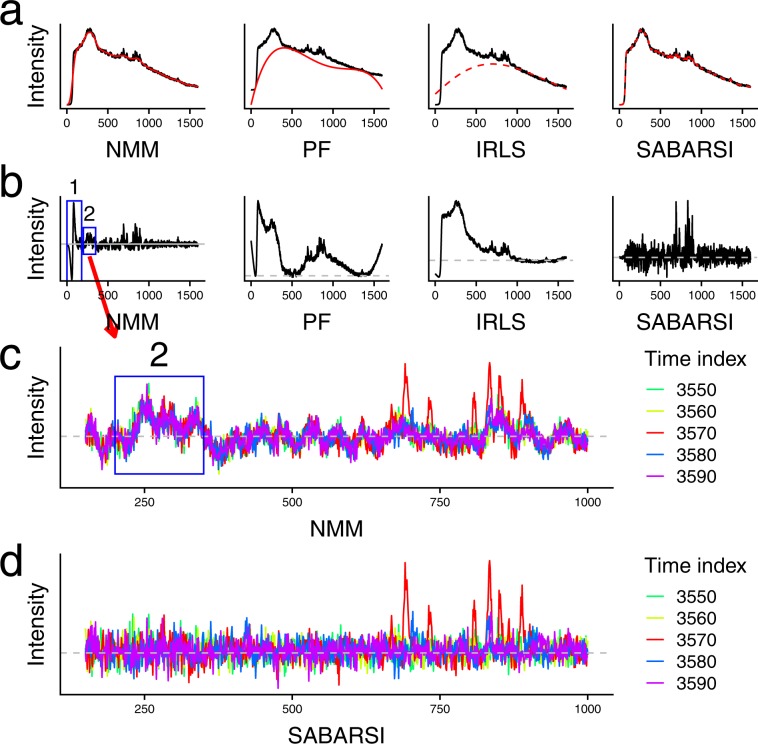
Performance of four different BCMs on the spectrum of riboflavin. (**a**) The original spectra (black lines) and the estimated backgrounds (red lines) by NMM, PF, IRLS, and SABARSI. (**b**) the corresponding background-corrected spectra (black lines). The two blue boxes in the leftmost figures highlight the two regions where NMM performs poorly. (**c**) Background-corrected spectra by NMM at four different time points, 3,550, 3,560, 3,570, 3,580, and 3,590. The blue box corresponds to the second blue box in (**b**). Apparently, these are backgrounds that have not been successfully removed. (**d**) Background-corrected spectra by SABARSI at the same set of four different time points. With SABARSI, the background has been removed thoroughly, highlighting the true signals (red lines in the 650~900 frequency range).

 Figure 2a,b show the results of background removal for the spectra of riboflavin in the first replicate using PF, IRLS, NMM, and SABARSI. The signal of riboflavin appears at time point 3,570 in the first replicate, and the results for the other B vitamins and/or other replicates are similar and not shown. Figure 2a shows the original spectra (black curves) and the estimated backgrounds (red curves) by the four methods, and Fig. 2b gives the background-removed spectra (black curves) generated by the four methods. Clearly, PF and IRLS fail to track the overall trend of the spectra closely and do not remove a significant proportion of background. NMM tracks the spectra much more closely than PF or IRLS, demonstrating the power of nonparametric methods. However, the steep positive and negative peaks at the leftmost region of the background-removed spectra (shown as the blue box 1 in the leftmost subfigure of Fig. 2b) are apparently mostly background. In fact, these peaks are actually stronger than the true signals (in the 650~900 frequency range), causing difficulties in identifying the true signals by this analysis. SABARSI clearly outperforms the other three methods by tracking the spectra closely and precisely, including the rapid fluctuation where NMM substantially failed.

Closer scrutiny of other regions gives us more evidence of the incomplete removal of background by NMM. In Fig. 2c, we plot the background-removed spectra from NMM for five different time points: 3,550, 3,560, 3,570, 3,580, and 3,590. We exclude in Fig. 2c the blue box 2 region in Fig. 2b so that other regions can be read more clearly. The first observation is the red peaks in the 650~900 frequency range. Undoubtedly these peaks are signals, since signals are typically Gaussian-shaped peaks of limited width, and they come and go and, thus, last a limited period of time. These peaks are actually signals from riboflavin. In contrast, noises are random fluctuations. Looking at one frequency channel, the noise should be positive at some time points and negative at others. Then, we discover a problematic feature of the background-removed spectra generated by NMM analysis: in regions that do not seem to have signals (regions other than the 650 ~ 900 frequency range), the fluctuations largely agree across time. For example, in the blue-box region, all the values are positive in all the five time points. Since no signals last that long, these values must include unremoved background. These unremoved background peaks have a similar magnitude as the true signals. In contrast, no such regions are present in the background-removed spectra generated by SABARSI (Fig. 2d). Except for the known signals in the frequency range 650 ~ 900, all other regions are just like white noises. Also, these noises have much smaller magnitudes than peaks from the riboflavin, making the true signals stand out.

These results demonstrate a much superior performance using SABARSI compared to using other methods that consider one spectrum at a time. However, the methods tested do not take into consideration the shape change of the background over time. In the next section, we compare SABARSI’s performance with another method, the constant-background correction method (“CBC”), that uses multiple spectra for background removal. Different from SABARSI, CBC assumes that the shape of background does not change over time.

### Background removal on the three vitamin dataset: comparison with CBC

**Figure 3:**
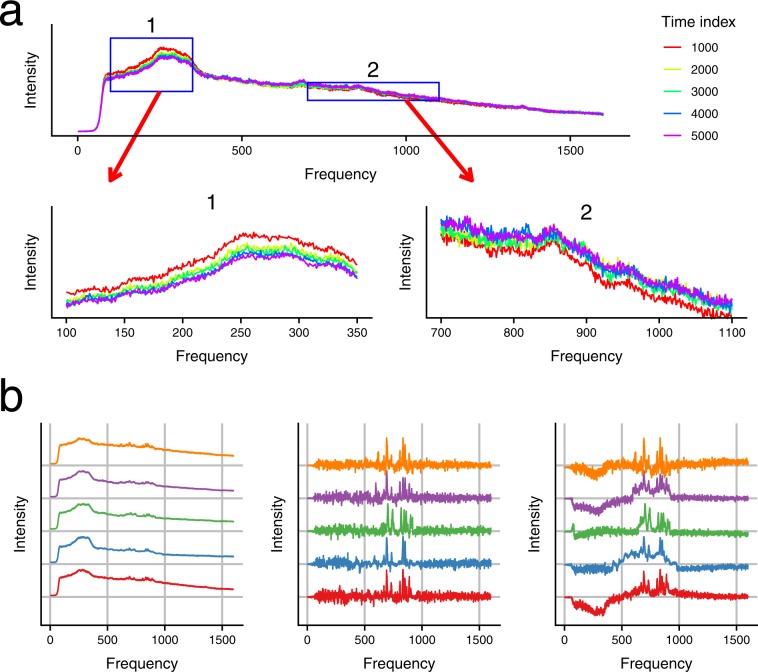
Change in the shape of backgrounds generated by SABARSI and CBC. (**a**) Spectra at five different time points (represented by different colors). Each spectrum is scaled by its average intensity to facilitate the comparison of the shape. Zoomed-in regions of spectrum fragments marked in the blue boxes are shown in the second row. (**b**) From left to right, the three plots show the original unprocessed spectra of riboflavin, background-removed spectra by SABARSI, and background-removed spectra by CBC, in five technical replicates (from top to bottom).

Analysis by CBC first scales each spectrum by the mean intensity of all frequency channels and then uses the average spectrum of all time points as the background. Unfortunately, we found that its key assumption, that the shape of the background does not change over time, is not true for any of our data. As an example, we show five spectra from time points 1,000, 2,000, 3,000, 4,000, and 5,000 in the first replicate, represented by different colors (Fig. 3a). In this figure, each spectrum has been scaled by its mean intensity. If the shape of the background does not change, then the lines of different colors should align with each other perfectly, except for small random deviations due to noise. However, the lines apparently diverge from each other in a systematic, non-random way. Background in the low frequency range (blue box 1 in the top figure, zoom-in view at the bottom left) decreases with time, and background in the middle-to-high frequency range (blue box 2 in top figure, zoom-in view at the bottom right) increases with time.

These violations of the constant background assumption lead to inferior performance in background removal. For example, Fig. 3b compares the background-removed spectra of riboflavin obtained by CBC and SABARSI in five technical replicates. While the background-removed spectra of riboflavin generated by SABARSI have bumps of highly consistent shapes across replicates, strong distortions are generated by CBC. Similar differences in performance of CBC and SABARSI are observed in the spectra for thiamine and folic acid, as shown in Figs. S1 and S2.

### Signal identification and comparison on the three vitamin data

Following background removal, we investigated the time indices of signature signals detected by SABARSI. Table 1 shows the pre-known (experimentally predetermined) time periods for the signals of three B vitamins and the corresponding time indices of signature signals given by SABARSI. For all three B vitamins in the five replicates, the time indices of signature signals given by SABARSI lie within the pre-known signal windows. Note that the pre-known time periods are very short, typically smaller than four time points. This is strong evidence that SABARSI identifies signals of interest reliably and accurately.

**Figure 4:**
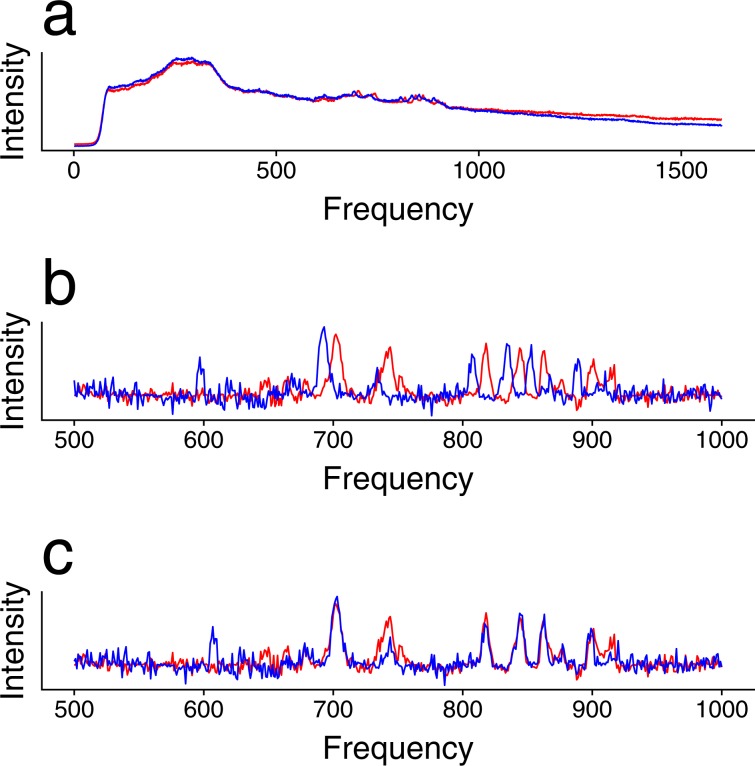
Spectra and signals of riboflavin from replicate 3 (blue) and replicate 4 (red). (**a**) Two spectra are scaled by their average intensities to be comparable in the same intensity scale. The fragments of signals in the frequency region 500~1,000 before (**b**) and after (**c**) the optimal frequency shift. The correlation coefficient of the two lines increases from 0.025 to 0.719 after the shift.

In signal comparison, we matched the signals of three B vitamins across five technical replicates with our novel similarity metric. Especially because one replicate has a significant shift of frequency channels, the signals in this replicate cannot be matched with those in other replicates with ordinary similarity metrics (e.g., Pearson’s correlation coefficient without considering the frequency shift). Figure 4 compares the spectra of riboflavin before (Fig. 4a) and after (Fig. 4b) background removal from the third (in blue) and fourth (in red) replicates, where Fig. 4c compares signals after one is shifted by the optimal number of channels given by our similarity metric. The overlapping of bumps is significantly improved after the frequency shift, and the correlation coefficient increases from 0.025 to 0.719. Comparison of the signals for thiamine and folic acid gives similar observations, as shown in Fig. S3.

### Signal identification and comparison on the tumor lysate data

The three vitamin dataset is a completely supervised dataset with only a few known analytes, while the tumor lysate contains hundreds to thousands of different molecules, most of which are unknown to us. Consequently, the SERS spectra of the tumor lysate changes even more substantially, making the background removal and signal identification more challenging. Here we show the effectiveness of SABARSI on such complicated data in identifying the spiked reference molecule.

**Figure 5:**
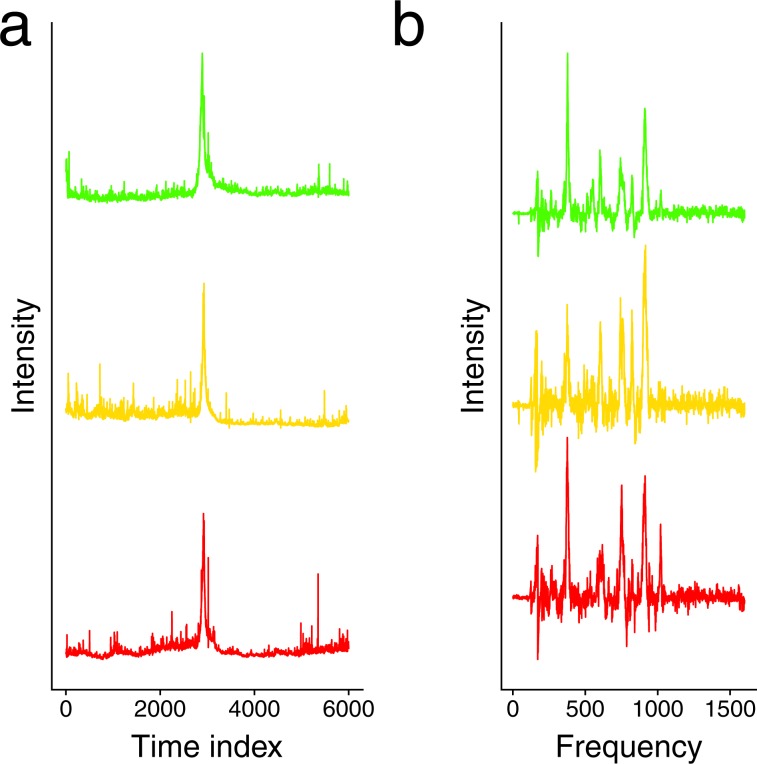
Identified signals corresponding to the spiked reference molecule. (**a**) The average intensities of spectra at different time points in three replicates of the tumor lysate data. The largest intensity that corresponds to the spiked reference molecule appears at time point 2,919, 2,929, and 2,893, respectively. (**b**) The signals identified in the three replicates that correspond to the spiked reference molecule. The pairwise Pearson’s correlations for the three signals are around 0.8.

 Figure 5a shows the average spectrum intensities at different time points. In all three replicates, a group of strong signals come right before 3,000, corresponding to the reference molecule that was spiked with high concentration. Since the strong signal lasts for a relatively long time in this data, we chose a relatively large window size for time, 150, to remove the background and then identified the signal with the highest intensity in each replicate. These three signals appear at 2,919, 2,929, and 2,893 time points in three replicates respectively, and, as Fig. 5b shows, they have very similar shapes (pair-wise Pearson’s correlation coefficients around 0.8). This again shows that the signals extracted by SABARSI are highly reproducible across replicates.

## Discussion

SERS technology provides the opportunity to identify analytes within complex mixtures of metabolites, and we have developed a statistical approach to remove the background from SERS spectra, identifying signals of interest, and measuring the similarity between signals. Compared with three popular BCMs and a constant-background method on a three vitamin dataset, our approach showed the most superior performance. Also, SABARSI successfully identified the spiked reference molecule in the complex tumor dataset.

SABARSI divides spectra into time-frequency blocks for background removal. This procedure involves two window-size parameters. We have conducted studies on the effect of window sizes and also give suggestions on how to choose them. Generally, the more rapidly the background changes with time, the smaller the window sizes should be. Overly large window sizes incompletely remove background, while overly small window sizes remove part of the signals. However, overall SABARSI is not sensitive to the choice of window sizes. For example, on the three vitamin dataset, the signals of three B vitamins barely change under window sizes 50, 100, and 200. Therefore, we expect the default choice in our SABARSI program to work well for a large variety of SERS datasets.

In the Results section, we have presented the comparison of SABARSI with four existing BCMs: NMM, PF, IRLS, and CBC. We have also compared SABARSI with three other BCMs: continueous wavelet transform^38^ (CWT, implemented in R package “baselineWavelet”^38^), Fourier transform filtering^39^ (FFT, implemented in R package “baseline”^33^) and asymmetric least squares^40^ (ALS, implemented in R package “baseline”^33^). These three methods also consider one spectrum at a time, just like NMM, PF, and IRLS. Figure. S4 shows their background-removed spectra of riboflavin in the three vitatmin dataset. Comparing with the lines shown in Fig. 2b, it is clear that these three methods also fail to remove significant amounts of the background, just like the other three BCMs that also consider one spectrum at a time.

There are many different techniques for spectroscopy (e.g.^41–44^). Although SABARSI is motivated by SERS, it should be appropriate, with minor modifications if needed, for any experiment where multidimensional data (spectrum versus time) has a time variant background in the spectral dimension. For example, SABARSI would straightforwardly apply to surface-enhanced resonant Raman scattering (SERRS) and ordinary Raman spectroscopy as described. In SERRS, the Raman signals are often more intense, which minimizes the need for background correction. SABARSI shines where the signals are small in magnitude compared to the background. The algorithm should translate straightforwardly to correct for fluorescence backgrounds, which are notoriously problematic in ordinary Raman spectroscopy. Applying SABARSI to Raman optical activity (ROA) will require some modification, which we leave as future work, as the signal of ROA is already a difference in intensity of left and right circularly polarized light, which can produce positive and negative features.

Recently, machine learning approaches^45–49^, especially neural networks, have been popular for SERS data analysis. There are several major differences/advantages of our SABARSI approach over machine learning approaches. First, machine learning approaches often use background-removed data; thus, the background-removal part of SABARSI provides robust preprocessing that may further boost the performance of machine learning approaches. Second, a key advantage of SABARSI is that it preserves the spectrum detected for further analysis. Machine learning approaches focus on assigning signals to classes or quantifying the signals, but the actual spectroscopic signals are not preserved in the treatment. Our approach enables traditional spectroscopic analysis on the samples. Third, our approach facilitates the use of SERS with chromatography, which has been challenging in the past. And, last but not least, machine learning approaches require a large amount of training data, e.g., data with known components and/or concentrations, while SABARSI can be used in an unsupervised manner.

We have made SABARSI publicly available as an R package named sabarsi on CRAN (https://cran.r-project.org/web/packages/sabarsi/index.html).

## Methods

### Animals and breast tumor lysates

Mice used in this study were maintained under pathogen-free conditions in the University of Notre Dame Freimann Life Sciences animal facility. Animal experiments were conducted in accordance with the University of Notre Dame Institution Animal Care and Use Committee guidelines after IACUC approval (protocol # 15-10-2724 and 18-11-5000). Breast tumors derived from MMTV-Wnt1^50^ mice were collected and used for this study. For this study, one tumor from an MMTV-Wnt1 mouse was used to generate the lysate used for technical replicates in this study. The tumor was lysed by first grinding it with mortar and pestle in liquid nitrogen and then resuspending it into three times its volume of lysis buffer (10 mM Tris HCl, pH 7.6, 5 mM EDTA, and 120 mM NaCl). The sample then was lysed using a sonicator for 10 second lysis, pause, and repeat for one minute. The sample was then centrifuged at 14,000 × g for 5 min, and the supernatant was collected. From the supernatant, a small sample was used to determine the protein concentration by Bradford assay using a standard curve of BSA. The protein concentration of the lysate was 5.79096 mg/mL. From the remaining supernatant, samples were prepared in 1 mL mixtures of 300 *μ*L lysate +700 *μ*L methanol, incubated at  −20C for one hour to precipitate, and then centrifuged at 14,000 × g to remove proteins. The remaining supernatant was then used for SERS. 100 *μ*L of samples (in methanol) were dried at room temperature using SpeedVac and then resuspended in water with 0.1% acetic acid. Because 2-Amino-3-pyridinol can produce stable and intense SERS signals, we selected it as the reference molecule and spiked 287 *μ*M of it into the tumor lysate.

### Overview of SABARSI

As illustrated in Fig. 1, SABARSI consists of four steps: background removal, signal detection, signal integration, and cross-experiment comparison. Novel statistical methods are proposed for each step. The first step is to remove the strong background from the original SERS spectra to obtain background-removed spectra, which consist of random noises and signals. For signal identification, a signal detection algorithm is applied to distinguish signals from noises and to give a set of time indices for the signals. In practice, many consecutive signals are highly similar and likely to come from the same nanoparticle. We integrate each group of concatenated signals to maintain a signature signal of them. In signal comparison, the minor mistake in wavenumber alignment may cause the signals to shift a few frequency channels in an experiment, which substantially decreases the correlation coefficients between identical signals across experiments. To address this variability, we here propose a new similarity metric to match identical signals while accounting for the potential shifts.

### Background removal

The background removal algorithm of SABARSI addresses the following observations and concerns. First, the shape of background along frequency channels can change steeply, and thus no smoothing should be applied along the frequency. Second, the shape of background changes over time; this change is typically slower but may trend differently on different frequency regions (See Fig. 3a). The algorithm is described in Algorithm 1.

First, the original matrix of spectra is divided into time-frequency blocks by taking fixed-size windows at both the time domain and the frequency domain. For instance, a dataset with 5,000 spectra and 1,600 frequency channels will be divided into 50 × 16 blocks when the window sizes in time and in frequency are both set to 100, and the first block, for example, contains the frequency channels from 1 to 100 in the first 100 spectra.

Next, within each time-frequency block, the fragments of spectra at different time points are scaled by their average intensities. This scaling removes the difference in the overall intensities and keeps only the shape. This shape is then captured by taking a pointwise median within the block. This median is taken over the time domain for every individual frequency channel and will not result in any smoothness on the frequency domain. Since median is used instead of mean, the signals, if present, will have virtually no effect on the estimation of the shape, and this shape reflects the shape of the background. Finally, the background (shape) is projected on each spectrum, and this projection is removed to give the background-removed spectrum. Algorithm 1 gives the whole algorithm for background removal.

Algorithm 1 for background removal is described as follows:

- Input: original SERS data in *T* time points and *W* frequency channels, which is given as a matrix $X={({X}_{ij})}_{T\times W}$, window size in the time domain *w*_*T*_, and window size in the frequency domain *w*_*F*_.
- Output: a matrix of background-removed spectra $Y={({Y}_{ij})}_{T\times W}$.

1. Segment time and frequency dimensions evenly by the corresponding window sizes to obtain *n*_*T*_ × *n*_*F*_ time-frequency blocks, where *n*_*T*_ = *T*∕*w*_*T*_ and *n*_*F*_ = *W*∕*w*_*F*_. Denote the fragments of spectra in each block by a matrix ${X}^{* }={({X}_{ij}^{* })}_{{w}_{T}\times {w}_{F}}$.
2. Scale each spectrum fragment at a time point, ${X}_{i\cdot }^{* },i\in \{1,2,\cdots ,{w}_{T}\}$, by its average intensity ${X}_{i\cdot }^{^{\prime} }={X}_{i\cdot }^{* }/mean({X}_{i\cdot }^{* })$. Then estimate the background for this block $B=({B}_{1},\cdots ,{B}_{{w}_{F}})$ in a pointwise manner by ${B}_{j}=median\{{X}_{ij}^{^{\prime} },1\le i\le {w}_{T}\}$. Note that median, instead of mean, is used to make the estimate robust to the possible presence of signals.
3. For each spectrum fragment in the block, calculate its pointwise projection vector onto the background ${P}_{i}=({P}_{i1},\cdots ,{P}_{i{w}_{F}})$ by ${P}_{ij}={X}_{ij}^{* }/{B}_{j}$. Then take the *q*’th (*q* = 40 was used in this paper) percentile of the values in *P*_*i*_ as an overall scaling factor and denote it by *Q*_*i*_. Finally, remove the estimated background at the original intensity scale by ${Y}_{i\cdot }={X}_{i\cdot }^{* }-{Q}_{i}\cdot B$.

### Signal detection

Background-removed spectra consist of signals of interest as well as random noises. Noises typically have relatively low magnitudes, and/or their values alter rapidly between positive and negative values. Signals, on the other hand, are usually positive and look like a set of bumps, which are defined as consecutive positive sections with relatively high magnitudes. Based on this, we have a mathematical definition (shown in Algorithm 2) that depends on three cutoffs: a cutoff for statistical significance that controls the false positive findings measured by false discovery rate (FDR)^51,52^, a cutoff for practical significance that controls the minimum magnitude of signals compared to the noise, and a cutoff of the length of the bump. The last cutoff is introduced based on the observation of presence, although rare, of sharp peaks with large magnitude but minimal length in frequency domain. These peaks are speculated to be due to cosmic rays^53^, and a length cutoff effectively rules them out. The whole algorithm is shown in Algorithm 2.

Algorithm 2 for signal detection is described as follows:

- Input: A matrix of background-removed spectra *Y* obtained from Algorithm 1. A cutoff *α* for the relative intensities of signals and an FDR cutoff *β*, a cutoff *γ* for bump length.
- Output: A set of time indices of signals, denoted by *t*.

1. For a background-removed spectrum, *Y*_*i*⋅_(*i* = 1, ... , *T*), estimate the standard deviation of noises *σ*_*i*_ by ${\widehat{\sigma }}_{i}=k\cdot median\{| {Y}_{ij}| ,j=1,\cdots W\}$, where *k* = 1∕(Φ^−1^(0.75)), and Φ^−1^ is the inverse cumulative function of the standard normal distribution. This estimate that uses MAD (median absolute deviation)^54^ is highly robust to the possible presence of signals.
2. Calculate the p-value for frequency channel *j* of *Y*_*i*⋅_ by
  $${p}_{ij}=\left\{\begin{array}{ll}2\times \Phi (-\frac{| {Y}_{ij}| }{{\widehat{\sigma }}_{i}}) & \,{\rm{if}}\,{Y}_{ij} > 0{\rm{,}}\,\\ 1 & \,{\rm{otherwise,}}\,\end{array}\right.$$
  where Φ is the cumulative function of the standard normal distribution. Then convert p-values (*p*_*i*1_, ... , *p*_*iW*_) into (*F*_*i*1_, ... , *F*_*iW*_), where *F*_*ij*_ is the FDR of the frequency channel *j* in spectrum *i*.
3. Find all bumps in *Y*_*i*_, where a bump is defined as a consecutive region of frequencies on which the magnitude satisfies *Y*_*ij*_ > *α* and *F*_*ij*_ < *β*. Let *L*_*i*⋅_ be a vector that records the length of bumps in *Y*_*i*⋅_. If max{*L*_*ij*_, *j* = 1, ⋅ , *W*} ≥ *γ*, claim that spectrum *Y*_*i*⋅_ has at least one signal and add its time index *i* into set *t*. Otherwise, claim *Y*_*i*⋅_ as a spectrum without any signal. Repeat this procedure for all background-removed spectra. Finally, the time index set *t* contains all the time indices that have at least one signal.

### Merging concatenated signals

The arrival of a type of analyte typically occupies multiple consecutive time points. Signals at these time points typically show similar shapes but of different strengths (e.g. first intensify and then fade). We use similarity in shape, measured by Pearson’s correlation coefficient, to judge whether signals consecutive in time come from the same type of analyte. If they do, then we only keep the signal with the strongest strength as the signature signal of this type of analyte. See Algorithm 3 for a detailed description.

Algorithm 3 for signal integration is described as follows:

- Input: A matrix of background-removed spectra *Y* (obtained from Algorithm 1), a time index set $t=\{{t}_{1},\cdots ,{t}_{{n}_{t}}\}$ (obtained from Algorithm 2, where *t*_*k*_ < *t*_*k*+1_, 1≤*k*≤*n*_*t*_, and *n*_*t*_ is the total number of time points with detected signals), and a threshold *ϕ* for similarity.
- Output: A set of time indices of signature signals, denoted by t^*^.

1. Add *t*_1_ to t^*^ and start from *k* = 2. If *t*_*k*_ = *t*_*k*−1_ + 1, the two signals are consecutive, and go to Step 2. Otherwise, go to Step 3.
2. Measure the similarity of two signals by Pearson’s correlation coefficient. If $Cor({Y}_{{t}_{k-1}\cdot },{Y}_{{t}_{k}\cdot }) > \phi$, go to Step 4. Otherwise, go to Step 3.
3. Add *t*_*k*_ to set t^*^. Continue to Step 1 for *t*_*k*_ and *t*_*k*+1_.
4. Let *S*_*k*−1_ and *S*_*k*_ denote the strength of signals in ${Y}_{{t}_{k-1}\cdot }$ and ${Y}_{{t}_{k}\cdot }$, where *S*_*k*_ is calculated as the median of signal magnitude in ${Y}_{{t}_{k}\cdot }$. If *S*_*k*_ > *S*_*k*−1_, then substitute *t*_*k*−1_ with *t*_*k*_ in set t^*^. Continue to Step 1 for *t*_*k*_ and *t*_*k*+1_.

### Similarity metric for cross-experiment comparisons

We propose a similarity metric of signals to deal with the possible shift along the frequency channel across experiments. This shift is typically less than ten frequency channels but can cause a substantial decrease in the similarity of signals when Pearson’s correlation coefficient is used directly. To account for this shift, we shift one signal by every possible number of frequency channels and calculate the Pearson’s correlation coefficient after the shift. The largest Pearson’s correlation coefficient is used as the similarity metric. Also, when calculating the Pearson’s correlation coefficient of a pair of signals, we only consider the informative section (the union of frequency ranges where signals occupy) in order to eliminate the influence of noises. For example, if signal A lies in the frequency range (400, 600), and signal B lies in the frequency range (500, 650), then the informative section is the frequency range (400, 650).

## Supplementary information

Supplementary Information.
