# Supplementary material for: A Statistical Approach of Background Removal and Spectrum Identification for SERS Data
Source: Sci Rep. 2020 Jan 29;10:1460. doi: 10.1038/s41598-020-58061-z (PMC6989639; doi:10.1038/s41598-020-58061-z)
Supplement: Supplementary file 1 — Supplementary Information. [file 41598_2020_58061_MOESM1_ESM.pdf]

# Supplementary Materials for A Statistical Approach of Background Removal and Spectrum Identification for SERS Data

Chuanqi Wang[1], Lifu Xiao[2], Chen Dai[3,4], Anh H. Nguyen[3], Laurie E. Littlepage[3,4], Zachary D. Schultz[2,3,4], and Jun Li[1,4,*]

[1]*University of Notre Dame, Department of Applied and Computational Mathematics and Statistics, Notre Dame, IN 46556, United States*
[2]*The Ohio State University, Department of Chemistry and Biochemistry, Columbus, OH 43210, United States*
[3]*University of Notre Dame, Department of Chemistry and Biochemistry, Notre Dame, IN 46556, United States*
[4]*Harper Cancer Research Institute, South Bend, IN 46617*
[*]*Correspondence and requests for materials should be addressed to J.L. (email: jun.li@nd.edu)*

# Experiments

## Reagents and materials

Acetonitrile (HPLC grade, $\geq$ 99.9%), folic acid ($\geq$ 98%), methanol (HPLC grade, $\geq$ 99.9%), phosphoric acid (crystalline, $\geq$ 99.999%), riboflavin ($\geq$ 98%), sodium hexanesulfonate ($\geq$ 99%), sodium phosphate monobasic dihydrate (NaH2PO4·2H2O, $\geq$ 99%), sodium tetraborate decahydrate ($\geq$ 99.5%), and thiamine hydrochloride ($\geq$ 99%) were purchased from Sigma-Aldrich (St. Louis, MO). A Barnstead Nanopure filtration system was used for 18.2 M$\Omega$ cm nanopure water. Anodized aluminum oxide (AAO) filters with 0.1 µm pores were purchased from Whitman (Germany). Fused silica capillary (72 µm i.d., 143 µm o.d.) was purchased from Polymicro Technologies (Phoenix, AZ).

## SERS substrate preparation

A previously reported thermal evaporation method was used to synthesize Ag SERS-active substrates by evaporating Ag into AAO filters[1]. Removing the AAO filter with 0.1 M NaOH (3.5 h) generates the used SERS substrate. The substrates are glued to a standard microscope glass slide that has two predrilled holes (35 mm apart) for incorporation into the sheath flow SERS cell.

## Liquid chromatography for the three vitamin model system

The stock solution of riboflavin (111 µM) and folic acid (37 µM) were prepared by dissolving weighed amounts in water with 0.3% v/v ammonium hydroxide. Thiamine (79 µM) stock solution was prepared in water. All mixtures were prepared by mixing stock solutions then diluting with water. Final concentrations of the mixture were 18.8 µM thiamine, 8.8 µM folic acid, and 0.4 µM riboflavin. A $50 \times 0.3$ mm capillary C18 column packed with 3-µm particles (Gemini, Phenomenex) was used for the HPLC separation. All LC experiments were performed on a capillary-scale LC Packings Ultimate system with quaternary pump and UV-vis detector (254 nm) with a 5.0 µL injection loop controlled by the Ultichrom software (LC Packings). The two mobile phases consisted of 0.050 M sodium phosphate buffer (with 0.005 M sodium hexanesulfonate) at pH 3.0 and a mixture of 50/50 methanol/acetonitrile (MeOH/ACN) pumped at a flow rate of 2 µL/min. The mobile phase gradient consisted of: (a) 0-2.99 min, 100% phosphate buffer; (b) 3-6.99 min, steady decrease to 80% phosphate buffer; (c) 7-14.99 min, hold at 80% phosphate buffer; (d) 15-19.99 min, steady change to 0% phosphate buffer and 100% MeOH/ACN; (e) 20-25 min, run at 100% MeOH/ACN to return to column's original condition.

## Liquid chromatography for tumor lysate samples

Chromatographic separation was achieved using an Ultimate 3000 RSLCnano HPLC system (Thermo Fisher) with two C18 columns—one trap column (Thermo Fisher, $0.075 \times 20$ mm, 3 µm) and one separation column (Thermo Fisher, $0.075 \times 150$ mm, 2 µm). Mobile phases were A: water (0.1% acetic acid) and B: acetonitrile (0.1% acetic acid). Flow rate was 300 nL/min. For each LC separation, 1 µL of tumor lysate sample was injected. The separation runs at isocratic 20% B for 20 min.

## Raman Measurement

All measurements were performed on a lab-built Raman microscope as previously reported[2]. The system was equipped with a 632.8 nm HeNe laser, a spectrograph (Shamrock 303i, Andor) with a 600 g/mm grating, and an EMCCD (Newton 970, Andor). Excitation and collection of SERS signal were obtained through a 40x water immersion objective (Olympus, NA=0.8). The laser power measured at the sample was 1-1.5 mW. The acquisition times were 250 and 200 ms for three vitamin and tumor experiments, respectively.

## Sheath-flow SERS

The sheath-flow SERS cell used has been previously described to confine the analytes to the SERS active surface[3]. The cell consists of an FEP base place, the glass microscope slide with SERS active substrate, a channel (1.5-2 mm slit) defining silicone gasket, a coverslip, and a stainless steel top plate. The capillary is glued to the SERS active substrate roughly centered in the sheath-flow channel. Samples were introduced by connecting the SERS flow cell online to the outlets of the above-described LC systems by a bare fused silica capillary. The sheath flow is established through the sample inlet and outlet in the plastic base plate. At the optimal sheath flow to capillary flow ratio, hydrodynamic focusing of the sample can be achieved[3]. The sheath flow rates of 140 µL/min and 30 µL/min were used for the

three vitamin and tumor experiments respectively. These rates were regulated by a LabView (National Instruments, Austin, TX) controlled syringe pump.

# Supplementary figures

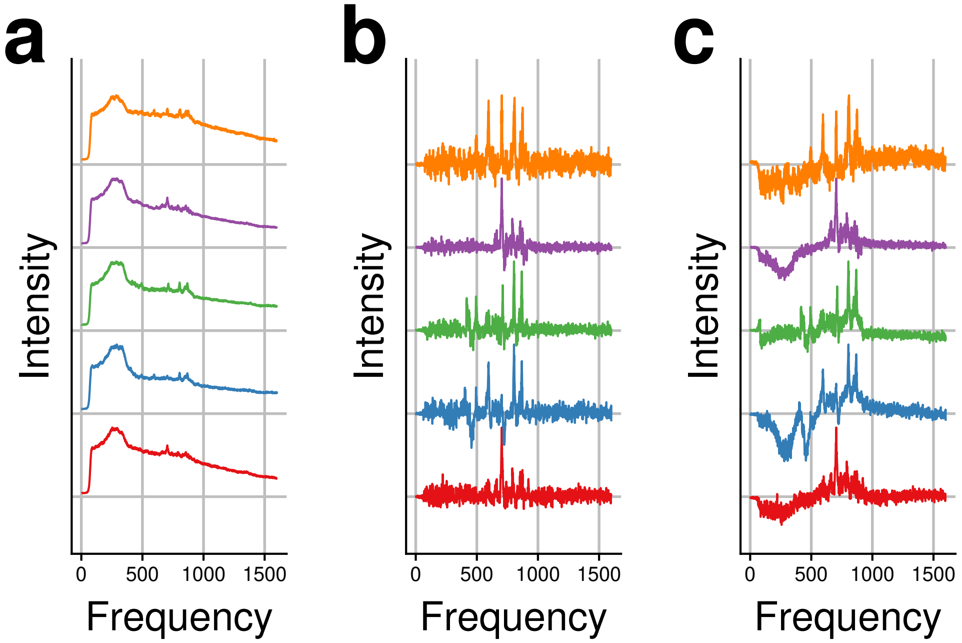


Figure S1: SERS spectra of thiamine **(a)** and background-removed spectra with SABARSI **(b)** and CBC **(c)** in five technical replicates.

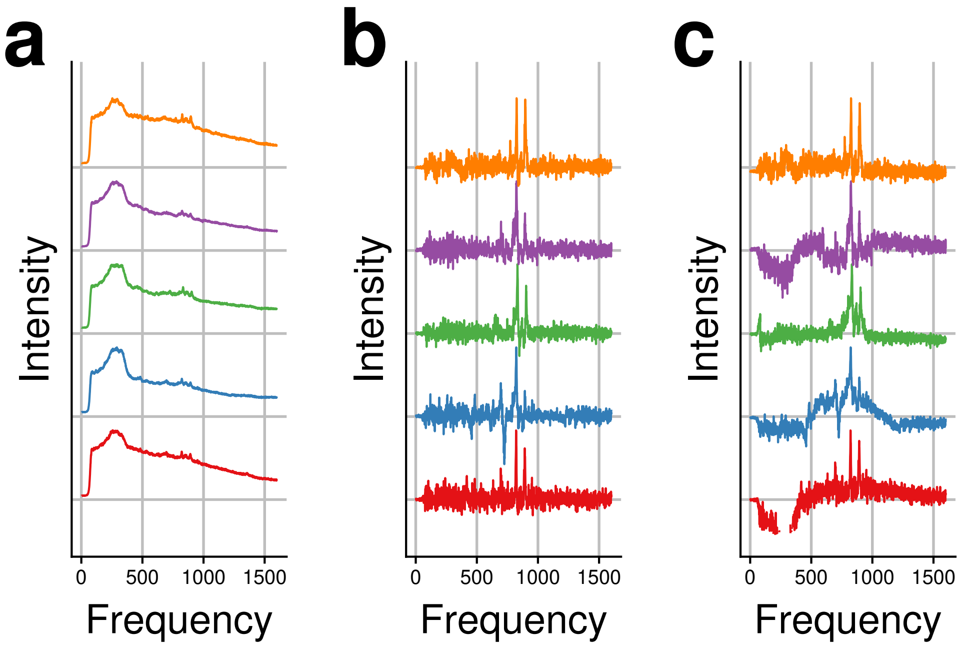


Figure S2: SERS spectra of folic acid **(a)** and background-removed spectra with SABARSI **(b)** and CBC **(c)** in five technical replicates.

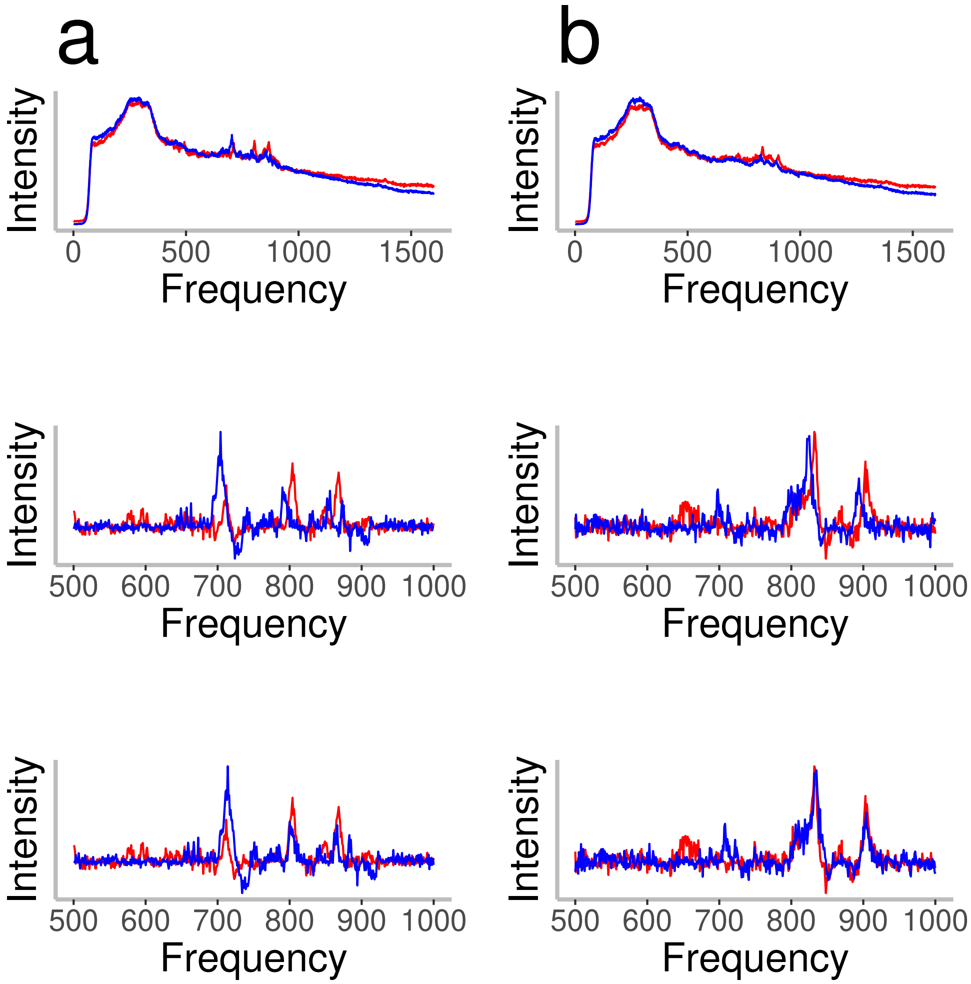


Figure S3: Frequency shift of the SERS Spectra for thiamine **(a)** and folic acid **(b)** from replicate 3 (in blue) and replicate 4 (in red). From top to bottom, the three rows are spectrum fragments with frequency channels within [500, 1000], corresponding signal fragments and shifted signal fragments.

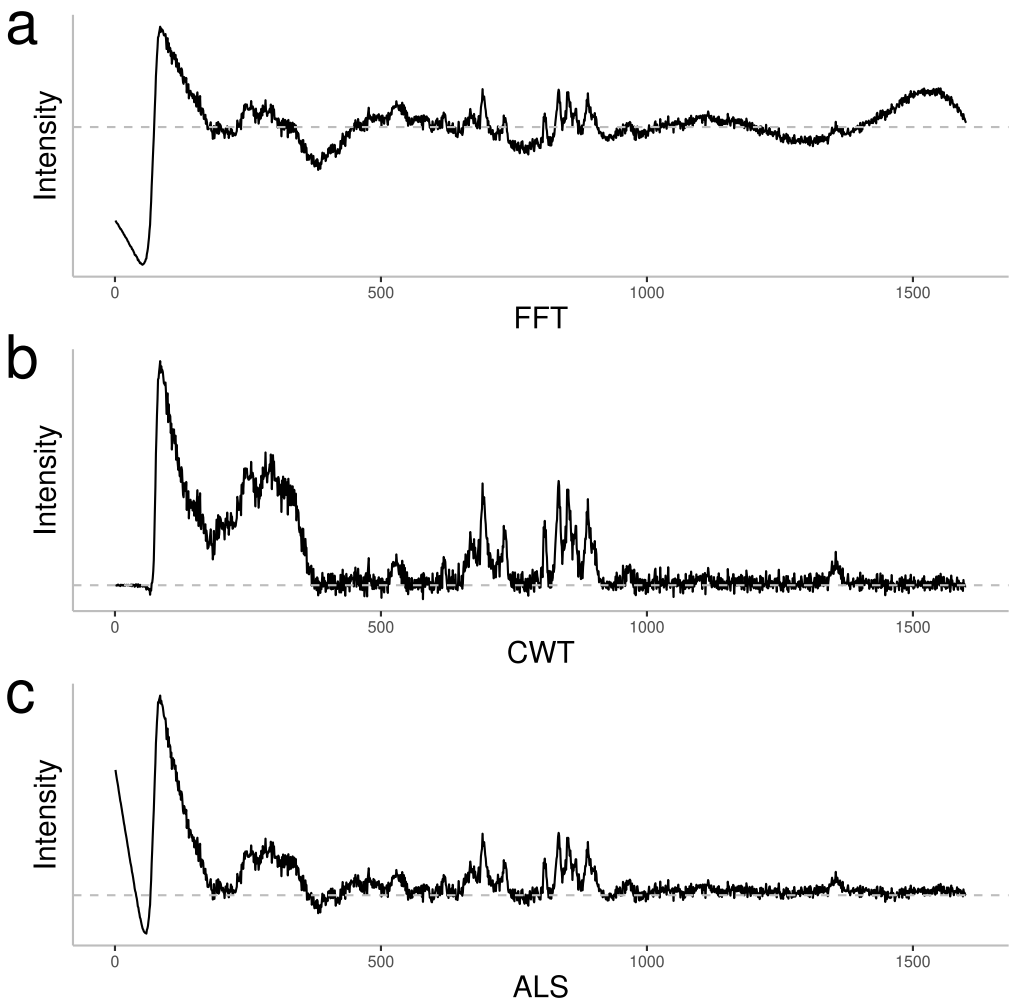


Figure S4: The background-removed spectra of riboflavin in the three vitatmin dataset by FFT **(a)**, CWT **(b)** and ALS **(c)**.

# References

[1] Steven M. Asiala and Zachary D. Schultz. Characterization of hotspots in a highly enhancing SERS substrate. *Analyst*, 136(21):4472–4479, November 2011.

[2] Matthew R. Bailey, R. Scott Martin, and Zachary D. Schultz. Role of Surface Adsorption in the Surface-Enhanced Raman Scattering and Electrochemical Detection of Neurotransmitters. *J. Phys. Chem. C*, 120(37):20624–20633, September 2016.

[3] Pierre Negri, Kevin T. Jacobs, Oluwatosin O. Dada, and Zachary D. Schultz. Ultrasensitive surface-enhanced Raman scattering flow detector using hydrodynamic focusing. *Anal. Chem.*, 85(21):10159–10166, November 2013.
